# Assessment of prevalence of Anxiety in adult population and development of anxiety scale: A study of 819 patients with anxiety disorder

**DOI:** 10.12669/pjms.37.2.2158

**Published:** 2021

**Authors:** Zaqia Bano, Maria Ejaz, Ishtiaq Ahmad

**Affiliations:** 1 Dr. Zaqia Bano, PhD. Department of Psychology, University of Gujrat, Gujrat, Pakistan; 2 Maria Ejaz, Department of Psychology University of Gujrat, Gujrat, Pakistan; 3 Dr. Ishtiaq Ahmad, FCPS. FRCS Department of Surgery, Al-Nafees Medical College, Isra University, Islamabad, Pakistan. University of Gujrat, Gujrat, Pakistan

**Keywords:** Anxiety scale, Behavior, Cognitive, Confirmatory factor analysis, Physical response subscales and prevalence, Reliability

## Abstract

**Objective:**

To develop an indigenous anxiety scale for adults and to assess the prevalence of anxiety among the adults.

**Methods:**

Descriptive explanatory research design was carried out from 1^st^ January 2018 to 31^st^ May 2019 at the Department of Psychology, University of Gujrat. The indigenous anxiety scale for adults was used for data collection. Scale consisted on three sub scales which are used to assess the cognitive symptoms, behavior and physiological symptoms through CFA value and alpha reliability. Sample of adequacy is .965, Confirmatory Factor Analysis value .914, alpha reliability .974 were taken as standard. In cognitive symptoms subscale’s alpha was 0.958, Behavior subscale .943 and Physiological symptoms subscale alpha was α .912.

**Results:**

The prevalence of anxiety in 20-29 years individuals exhibited 17.5% (male 38.8%; female 62.2) symptoms of anxiety. 30-39 Years people showed 12.9% (male =56.9%; female=43.1%) , 40-49 years individuals showed 16.5% of anxiety and in late adulthood 50-60 years old people showed highest level of anxiety 22.10% (male=68.3%; female= 31.7).

**Conclusion:**

The anxiety scale for adults is a reliable indigenous scale for measuring anxiety in adults. Further, the prevalence rate of anxiety in adult population is alarming indication and middle and late adulthood suffered more anxiety as compare to early adulthood.

## INTRODUCTION

Anxiety is a normal human emotion and it may be a result of stress. The pathological anxiety is defined as an excessive, unpredicted, unmanageable, and overwhelming emotional response to apparent and looming threat. Anxiety may be manifested by constant worry, tension and biological symptoms like palpitation, increase blood pressure and uneasiness.^1^,^2^ Anxiety disorder is the most prevalent mental health issue and significantly effects the personal, social and occupational life of the individuals. Furthermore, anxiety intensely influenced the quality of life of individuals and enhances the chances of heart problem, risk of suicide and other medical issues.^3^ A study conducted by Dhal et al for assessing anxiety in adults concluded that the males who suffered anxiety had poor marital relation, drug addiction, low intake of fruit and vegetables, low income and are at high risk of developing cardio vascular problems and Asthma.^4^

A large scale population survey by Bandelow and Michaelis reported that up to 33.7% of the population are affected by anxiety disorder during their life.^5^ The results indicated that the anxiety disorders are the most prevalent, disturbing, persistent, chronic, utmost disabling disorders and frequently comorbid with depression and substance abuse disorder.^6^ A recent British survey results indicated that almost 16% of the population suffering from pathological anxiety.^7^ According to a WHO report about 40 million Americans around the age of 18 and above are suffering from anxiety. It further explained that almost 27% old age people showed anxiety symptoms and among them 14% is experiencing severe anxiety disorder.^8^

A study by Ritchie and Roser observed that worldwide women suffered anxiety twice then males. In the overall population of the world, 170 million females (62% females) suffered anxiety disorder.^9^ Maya and colleagues^10^ also reported that women experienced more anxiety (63%) then males (40 % ). Among young and old age anxiety patients, the older adult patients show more symptoms of anxiety.^11^ Especially, adolescents at high risk and even in crises situation they are more prone to anxiety disorder, irritability and restlessness.^12^,^13^

To measure the anxiety level, so far there is no indigenous scale is developed or available in Pakistan. We are relying on the scales developed by the Western world that are having a totally different demographic, socio-economic, religious, environmental and cultural pattern from Pakistan. Moreover, these scales are not in our native language and neither covers the all aspects of our life patterns mentioned before. There is a dire need to develop a standardized scale covering all aspects in native language. This self-administered scale can be used in both clinical and nonclinical settings, local surveys and by social workers. Further, ASA will measure the cognitive, behavior and physiological aspects of anxiety and levels of anxiety as well. This study was conducted with an objective to develop an indigenous anxiety scale for adults and to assess the prevalence of anxiety among the adults.

## METHODS

This descriptive explanatory research design study was conducted at the Department of Psychology, University of Gujrat from 1^st^ January 2018 to 31^st^ May 2019. Ethics Committee meeting held on 23.10.2018 and approved by Advanced Studies & Research Board in meeting held on 12.02.2019, under Vide notification No. UOG/ASRB/Psychology/03/17677 dated March 1, 2019. A total of 819 participants from genders between the ages of 30 years were selected through convenient sampling technique from different areas of District Gujrat. Both genders, clinical and non-clinical population with anxiety disorder were included in the study. The individuals below the age of 18 years, having physical or intellectual disability and diagnosed patient with other psychiatric disorders were also excluded from study. Two scale Demographic information Form which `consisted of personal information, age, gender, education and birth order and newly developed anxiety scale for adults^14^ were used to measure anxiety in adults. After the proper permission of Institution authorities and Departmental research review committee the participants were approached in community and Hospital by the researcher. Before data collection confidentiality were ensured to the participants.

### Data analysis

In order to interpret the data IBM SPSS V-21, and AMOS Graphics was used. Descriptive statistics was used to measure the prevalence of the anxiety. Reliability analysis, correlation analysis and Dimension reduction was used for exploratory factor analysis. AMOS Graphics was used for the purpose of Confirmatory factor analysis.

## RESULTS

A total 819 participant’s between the age range of 30 years were assessed with a mean age of 20-60 years from both sex (male=473, female=346). Majority of the patients (n=143, 17.5%) were from 3^rd^ decade of life followed by 5^th^ decade i.e. 16.5% (n=135) as shown in Table-I.

**Table-I:** Frequency of age and sex distribution across various age groups (N=819).

*Age group*	*Gender*	*F*	*%*	*Total %*
(20-29) Years	Male	76	38.8	143(17.5 %)
	Female	120	62.2	
(30-39) Years	Male	107	56.9	106(12.9 %)
	Female	81	43.1	
(40-49) Years	Male	139	65	135(16.5 %)
	Female	75	35	
(50-60) Years	Male	151	68.3	181(22.10 %)
	Female	70	31.7	

***Note:*** f= stands for Frequency, %= Percentage of Gender.

In scale development, for measuring exploratory factor analysis factors were fixed on 5.00. After extraction of the items and Varimax Rotation final 45 Item remained on Anxiety Scale for Adults (ASA) (n=334). Cognitive symptoms of anxiety consisted 18 loaded items. Item 1(0.606), 2(0.683), 3(0.672), 4(0.587), 5(0.618), 6(0.666), 7(0.617), 8(0.523), 9(0.678), 10(0.608), 11(0.571), 12(0.658), 13(0.590), 14(0.562), 15(0.560), 16(0.560), 17(0.640) and 18(554). Whereas, on behavior symptom of anxiety sub-scale comprised 16 items as items no 19(0.489), 20(0.576), 21(0.613), 22(0.611). 23(0.710), 24(0.614), 25(0.591), 26(0.600), 27(0.629), 28(0.615), 29(0.623), 30(0.632), 31(0.605), 32(0.550), 33(0.78), and item no 34(0.586). Furthermore, Physiological symptoms of anxiety sub-scale consisted on 11 items. Item no 35 value was (0.625), 36(0.585), 37(0.629), 38(0.617), 39(0.684), 40(0.711), 41(0.689), 42(0.616), 43(0.615), 44(639), and 45(0.678). Sample adequacy of the ASA was highly adequate such as KMO was .967, Chi-Square 10155.416, df, 990 at 0.00 level of significance.

Overall Cronbach’s Alpha reliability of the Anxiety Scale for Adults was 0.974 (N=334). Further, reliability of the three subscales of ASA was highly significant such as Cognation Symptoms of anxiety was 0.958, Behavior Symptoms 0.943 and Physiological Symptoms subscale reliability was 0.912 .

Prevalence of severity of anxiety in both genders is shown in Table-II. Score ranges above 117 showed High level of anxiety, from 59-117, moderate and below 59 scores exhibited as low level of anxiety. Frequency of severity of moderate anxiety were highest ie 53.96% (n=442) whereas mild anxiety was found in only 5.98% (n=149) patients in both gender.

**Table-II:** Frequency of severity of anxiety among both gender (N=819).

*Severity of anxiety*	*Total N=819*	*Male n=473 (57.76 %)*	*Female n=346 (42.24 %)*

	*(%)*	*F(%)*	*F(%)*
Severe(Above117)	328(40%)	193(58.8%)	135(41.2%)
Moderate(59-117)	442(53.96%)	244(55.2%)	198(44.8%)
Mild(Below 59)	49(5.98%)	36(73.5%)	13(26.5%)

Frequency of severity of anxiety in both gender in different age groups is shown in Table-III. Severe anxiety were observed highest in 5^th^ (n=98,29.9%) and 6^th^ decade (n=85, 25.9%), whereas moderate severity of anxiety were observed in 3^rd^ (n=118, 26.7%) and 6^th^ decade (n=112, 27.6%) of life. The frequency of mild anxiety is almost same ie 2h8.6% in 3^rd^, 5^th^ and 6^th^ decade of life.

**Table-III:** Frequency of severity of anxiety among both Gender in different age groups (N=819).

*Age*	*Score*	*N(%)*
(20-29) Years	Severe	64 (19.5%)
	Moderate	118 (26.7%)
	Mild	14 (28.6%)
(30-39) Years	Severe	81 (24.7%)
	Moderate	100 (22.6%)
	Mild	7 (14.3%)
(40-49) Years	Severe	98 (29.9%)
	Moderate	102 (23.1%)
	Mild	14 (28.6%)
(50-60) Years	Severe	85 (25.9%)
	Moderate	112(27.6%)
	Mild	14(28.6%)

## DISCUSSION

The main objective of the current study was to develop a valid measure of anxiety for adults. Anxiety scale for adults (ASA) developed according to the beck model (Cognition, Behavior and Physiological symptoms). In exploratory factor analysis the factor were loaded on 0.5 level of significance and after loading remained 110 items. Exploratory factor analysis is important in the factor analysis process; exploratory factor analysis explored the variables and generates factors. This process provided the guideline about the number of factor.^15^ For testing the adequacy of the data KMO was .967 on .00 level of significance which was sufficiently adequate.^16^

Confirmatory factor analysis was conducted to confirm the factor given by EFA. CFA unlike EFA provide a complete system to check the testing model.^17^ A modification index was run to check the problematic items and increase the value of Comparative fit index (CFI). The RMESA values also tell the developer how well the model is fit.^18^ The value of Root mean Squared error of approximate is .51 which is less then 0.08 which showed the model is good fit. The value of RMESA should be ≤8 for model fitness and if it is ≤.05 then it means the model is closed fit and in this case the model is closed fit.^19^

The value of Turker Lewis index (TLI) is .910 which means the model is approximately fit. The value of TLI<.90 means that the model is inadequate fit. But in this case the value of TLI is greater then .90 so we can say that the model is fit and there is no need of substantial changes in the data. The value of GFI is .812 which is closed to .09 and shows that the model is closed to adequate fit. The range of GFI is between 0 to one and it will increase as the sample increase.^20^ The value of CMIN/DF is 1.882 which is less than three and show the fitness of model.^21^ Chi square test was used to check the goodness of the test the p is .00 which is less then .05. It showed that the model is satisfactory fit.^22^ he value of Comparative fit index (CFI) is .914 which is greater than .90 show that the model is satisfactory fit.^23^ So the result shows that the model is adequately satisfacstory.The reliability of the scale was (0.974) and the reliability of the three subscales cognitive (0.958), behavior (0.943 ) and physiological symptoms (0.912 ) were highly significant Research. Further, the value of Cronbach’s alpha should be between 0 to will be considered significant.^24^

Further, result showed the prevalence of anxiety was higher in males in middle and late adulthood as age ranges 30-39 years (56.9%); 40-49 years male showed higher anxiety symptoms ((65%), 50-60 years 68.3 %% as compare to females 43.1%, 35 % and 31.7 at the age of 30-39, 40-49 and 50-60 years of age. Whereas, in early adulthood from 20-29 years of age female exhibited higher anxiety 62.2% as compare to male as 38.8%. The prevalence of anxiety around the world varies 2.5 to 6.5 from country to country. Global estimation of anxiety disorder 2016 shows that 275 million people experienced anxiety. Among those peoples 62% were females and 38% were males^25^; the prevalence rate of anxiety over life time is 31%.^26^ Malathy Lyer explained^27^ 38 million (7.5%) Indian suffered anxiety disorder. Further, in Iran 12% males and 19.4% females experienced anxiety disorder.^.28^ Ministry of Health Iraq explained that anxiety disorder in Iraq was 13.8%.^29^ Literature reported that the prevalence of anxiety has significantly increased during last ten years in Pakistan, as 60% people exhibited the symptoms of anxiety.^30^ According to the 2017 report of Institute of Health Metrics and Evaluation (IHME), anxiety is a most disabling issue and affects around 34.0% of the population in Pakistan.^31^

In last decade anxiety has become hazardous problem in Pakistan. Although epidemiological studies of anxiety disorders are available. Current study estimation showed the prevalence of anxiety such as 328(40%) individual showed high, 442(54%) moderate level and 49(6%) people respond low level of anxiety. Among the gender females suffered from more anxiety in early adulthood whereas male experienced pathological anxiety in middle and late adulthood. One current study conducted in Sialkot is also in line that 89 (17.8%) University students showed severe anxiety and 46.8% exhibited extereme sever anxiety symptoms.^32^ Futhermore 14 years and above adolescents in Karachi suffered 11% anxiety disorders symptoms.^33^ Researcher^34^ have explained that when anxious person’s age increased the level of anxiety was also significantly increased.

### Limitations of the study

Data was collected in the District Gujrat, so the findings have the issue of generalizability. Current study focused on adolescent population and data was gathered only from institutions. Precipitating factors of anxiety in adolescents could not become the part of the study. The construct of ‘Anxiety’ was measured as a whole, although self-reported anxiety scale was comprised the three factors of anxiety those factors (Cognitive, behavior and physiological symptoms of anxiety) were not individually evaluated.

## CONCLUSION

The anxiety scale for adults is a reliable indigenous scale for measuring anxiety in adults. Further, the prevalence rate of anxiety in adult population is alarming indication and middle and late adulthood suffered more anxiety as compare to early adulthood.

### Author`s Contribution:

**ZB** conceived, designed methodology, statistical analysis, manuscript writing, & editing of manuscript. She is also responsible and accountable for the accuracy or integrity of the work.

**ME** did data collection, statistical analysis and responsible and accountable for the accuracy or integrity of the work.

**IA** did review, editing and final approval of manuscript.
